# Additive Inhibition of Reflex Bladder Activity Induced by Bilateral Pudendal Neuromodulation in Cats

**DOI:** 10.3389/fnins.2020.00080

**Published:** 2020-02-07

**Authors:** Katherine Shapiro, Natalie Pace, Tara Morgan, Haotian Cai, Bing Shen, Jicheng Wang, James R. Roppolo, William C. de Groat, Changfeng Tai

**Affiliations:** ^1^Department of Urology, University of Pittsburgh, Pittsburgh, PA, United States; ^2^School of Health and Rehabilitation Sciences, University of Pittsburgh, PA, United States; ^3^Department of Pharmacology and Chemical Biology, University of Pittsburgh, Pittsburgh, PA, United States; ^4^Department of Bioengineering, University of Pittsburgh, Pittsburgh, PA, United States

**Keywords:** pudendal, nerve, neuromodulation, bladder, cat

## Abstract

**Objective:**

To determine the inhibitory effect on bladder activity induced by bilateral pudendal neuromodulation.

**Methods:**

In 10 cats under anesthesia, two tripolar cuff electrodes were implanted bilaterally on the pudendal nerves for stimulation. A double lumen catheter was inserted into the bladder through the urethra to infuse saline and measure bladder pressure. During repeated cystometrograms (CMGs) pudendal nerve stimulation (PNS: 5 Hz, 0.2 ms, 5–15 min) was applied unilaterally or bilaterally at 1- or 2-times intensity threshold (*T*) for inducing anal sphincter twitching. PNS inhibition was indicated by the increase in bladder capacity measured by CMGs.

**Results:**

Unilateral PNS at 1T did not significantly increase bladder capacity, but at 2T significantly (*p* < 0.05) increased bladder capacity by about 30%. Bilateral PNS at 1T also failed to increase bladder capacity, but at 2T significantly (*p* < 0.05) increased bladder capacity by about 60%, indicating an additive effect induced by the bilateral 2T PNS. Unilateral 1T PNS did not enhance the inhibitory effect induced by contra-lateral 2T PNS.

**Conclusion:**

This study in anesthetized cats reveals that an additive inhibition of reflex bladder activity can be induced by bilateral pudendal neuromodulation, indicating that bilateral PNS might achieve better therapeutic efficacy in treating overactive bladder (OAB) than unilateral PNS.

## Introduction

Sacral neuromodulation is approved by FDA as an effective therapy for overactive bladder (OAB). However, this therapy is only successful in about 70% of OAB patients (van Kerrebroeck et al., 2007). The standard sacral neuromodulation therapy implants a single electrode lead unilaterally on one sacral S3 spinal root. Implantation of a second electrode lead on the sacral S3 spinal root on the other side is only performed when unilateral sacral neuromodulation fails to treat OAB effectively (Marcelissen et al., 2011). A clinical study showed that OAB patients who failed unilateral sacral neuromodulation could be treated effectively by bilateral sacral neuromodulation (Marcelissen et al., 2011). However, it is not clear whether the additional beneficial effect is caused by the stimulation delivered via the second electrode alone or by interaction between the left and right neuromodulation.

The mechanism underlying sacral neuromodulation is currently unknown. However, it is assumed that the efficacy of this neuromodulation is due in part to stimulation of afferent nerve fibers projecting into the pudendal nerve (Peters et al., 2005). Clinical studies showed that pudendal neuromodulation successfully treated OAB patients who failed sacral neuromodulation (Peters et al., 2005, 2010). Currently whether bilateral pudendal neuromodulation is more effective than unilateral pudendal neuromodulation is still unknown. Although many previous studies in both animals (Larson et al., 2011; Yecies et al., 2018) and humans (Peters et al., 2005, 2010) have investigated pudendal neuromodulation of bladder activity, there is no study investigating the interaction between bilateral pudendal neuromodulation. Whether bilateral pudendal afferent inputs can generate an additive inhibition of bladder activity or a synergistic inhibitory effect (i.e., greater than additive effect) is unknown. More importantly, whether ipsilateral pudendal afferent input that is ineffective in inhibiting bladder activity can potentiate the effect of contra-lateral pudendal afferent input in inhibiting the bladder is also unknown. Answering these questions is important for a better understanding of pudendal neuromodulation in the treatment of OAB. The micturition reflex is mediated by parasympathetic (pelvic nerve), sympathetic (hypogastric nerve), and somatic (pudendal nerve) pathways (Fowler et al., 2008). OAB could be caused by an abnormality in any of these 3 neural pathways. Due to this complexity the pathophysiological cause for OAB is currently unknown. However, pudendal neuromodulation has been proven to be effective in treating OAB (Peters et al., 2005, 2010) even though its mechanism of action is still unknown.

This study examined the influence of unilateral and bilateral pudendal nerve stimulation (PNS) on reflex bladder activity with the goal of establishing how the magnitude of neuromodulation is influenced by simultaneous inputs from bilateral pudendal nerves. In anesthetized cats, left and/or right pudendal nerves were electrically stimulated; and the evoked inhibition of bladder activity was evaluated to determine whether bilateral neuromodulation can generate stronger inhibition than unilateral neuromodulation and whether the combined effect is additive or synergistic. Addressing these issues could have an impact clinically on the decision to use unilateral or bilateral neuromodulation in treating OAB.

## Materials and Methods

### Surgical Protocol

A total of 10 cats (6 females, 4 males, 3–4.4 kg; Liberty Research, Waverly, NY, United States) were used in this study. The animals were anesthetized initially with isoflurane (2–5% in oxygen) during surgery and then switched to alpha-chloralose anesthesia (initial dose 65 mg/kg i.v. followed by supplemental doses as needed) during data collection. The right cephalic vein was catheterized for intravenous administration of fluid and drugs. A midline anterior cervical incision was used to access the airway, which was kept patent via tracheostomy. The right carotid artery was catheterized for monitoring arterial blood pressure. Oxygen saturation and heart rate were measured via a pulse oximeter (9847VNONIN Medical, Plymouth, MN, United States) attached to the tongue.

A laparotomy was performed and ureters were isolated bilaterally, cut and drained externally. A double lumen catheter was inserted into the bladder via a small cut at the proximal urethra and tied in placed by a suture. One lumen was connected to a pump to slowly infuse the bladder with saline. The other lumen was connected to a pressure transducer to measure pressure changes in the bladder. The pudendal nerve was isolated in the region of the sciatic notch bilaterally. Tripolar cuff electrodes (NC223pt, MicroProbe, Gaithersburg, MD, United States) were implanted around these nerves for stimulation. The muscle and skin were closed by sutures at the end of surgery. The animals were humanely euthanized at the end of the experiments under deep anesthesia.

### Experimental Protocol

Uniphasic rectangular pulses (5-Hz frequency, 0.2-ms pulse width) were used for PNS in this study. These parameters have previously been shown to be effective in inhibiting reflex bladder activity (Larson et al., 2011; Kadow et al., 2016). At the beginning of the study, the intensity threshold (*T* = 0.24–1.0 V) for PNS to induce observable anal sphincter twitch was determined. In this study the intensity threshold *T* was set as low as possible by adjusting the intensity with a 0.01 V resolution to induce a weak anal sphincter contraction that did not inhibit the bladder activity, which allowed the investigation of potential synergistic effect induced by bilateral ineffective neuromodulation or potentiation of an effective neuromodulation on one side by an ineffective neuromodulation on the other side.

**FIGURE 1:**
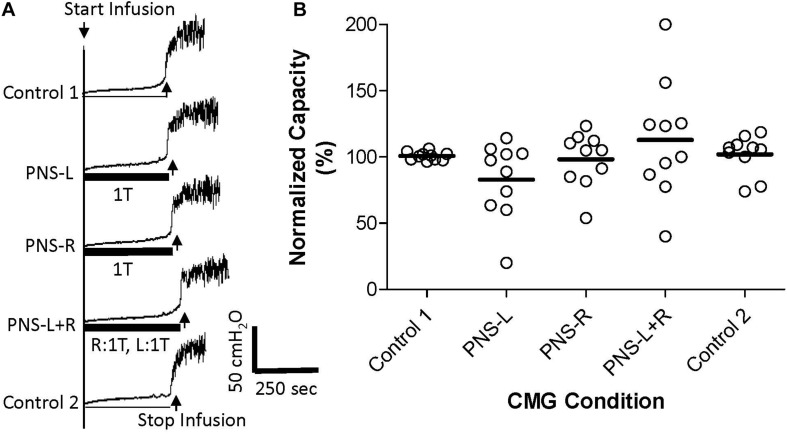
Effect of bilateral low intensity (1T) pudendal nerve stimulation (PNS) on bladder capacity. **(A)** Cystometrogram (CMG) tracings demonstrate bladder capacities during control conditions or during left PNS with 0.7 V (1T) intensity (PNS-L), right PNS with 0.45 V (1T) intensity (PNS-R), or bilateral 1T PNS (PNS-L + R). The thick black bar under CMG tracings indicates the PNS duration. **(B)** Summarized bladder capacity measured during different CMG conditions. The capacity was normalized to the Control 1. *N* = 10 cats. There was no significant difference in mean bladder capacity with unilateral or bilateral PNS at 1T intensity.

**FIGURE 2:**
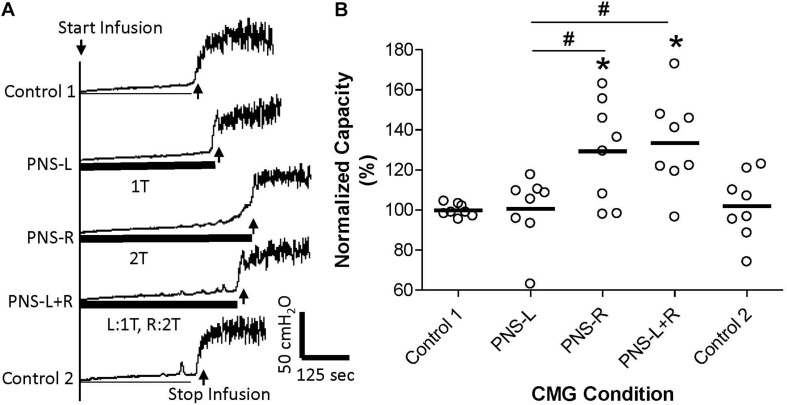
Effect of unilateral low intensity (1T) pudendal nerve stimulation (PNS) on contra-lateral high intensity (2T) PNS. **(A)** Cystometrogram (CMG) tracings demonstrate bladder capacities during control conditions or during left PNS with 0.7 V (1T) intensity (PNS-L), right PNS with 0.9 V (2T) intensity (PNS-R), or bilateral PNS with left 1T and right 2T (PNS-L + R). The thick black bar under the CMG tracings indicates the PNS duration. **(B)** Summarized bladder capacity measured during different CMG conditions. The capacity was normalized to the Control 1. *N* = 8 cats. *Indicates significantly (*p* < 0.05) different from Control 1 # indicates significantly (*p* < 0.05) different between the two conditions marked by the line.

**FIGURE 3:**
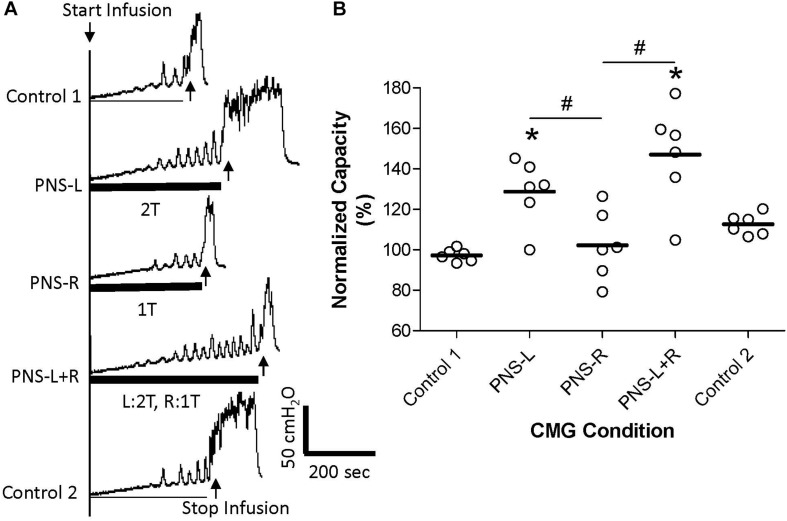
Effect of unilateral low intensity (1T) pudendal nerve stimulation (PNS) on contra-lateral high intensity (2T) PNS. **(A)** Cystometrogram (CMG) tracings demonstrate bladder capacities during control conditions or during left PNS with 1.4 V (2T) intensity (PNS-L), right PNS with 0.45 V (1T) intensity (PNS-R), or during bilateral PNS with left 2T and right 1T (PNS-L + R). The thick black bar under CMG tracings indicates the PNS duration. **(B)** Summarized bladder capacity measured during different CMG conditions. The capacity was normalized to the Control 1. *N* = 6 cats. *Indicates significantly (*p* < 0.05) different from Control 1. #Indicate significantly (*p* < 0.05) different between the two conditions marked by the line. There is no significant difference between PNS-L and PNS-L + R.

**FIGURE 4:**
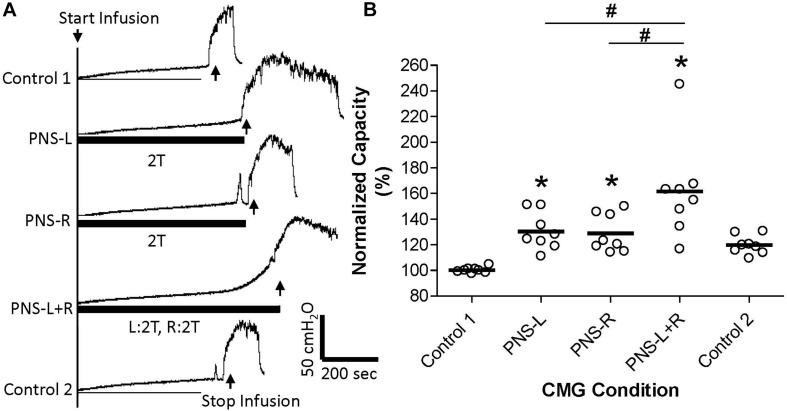
Effect of bilateral high intensity (2T) pudendal nerve stimulation (PNS) on bladder capacity. **(A)** Cystometrogram (CMG) tracings demonstrate bladder capacities during control conditions or during left PNS with 0.8 V (2T) intensity (PNS-L), right PNS with 1.0V (2T) intensity (PNS-R), or bilateral 2T PNS (PNS-L + R). The thick black bar under CMG tracings indicates the PNS duration. **(B)** Summarized bladder capacity measured during different CMG conditions. The capacity was normalized to the control 1. *N* = 8 cats. *Indicates significantly (*p* < 0.05) different from Control 1. #Indicate significantly (*p* < 0.05) different between the two conditions marked by the line.

Initially repeated cystometrograms (CMGs) were performed by slowly (1–2 mL/min) infusing the bladder with saline to determine the reproducibility of the control bladder capacity. Bladder capacity was defined as the minimal volume required to induce a reflex bladder contraction of large amplitude (>30 cmH_2_O) and long duration (>20 s). Then, four groups of CMGs illustrated in Figures 1–4 were performed sequentially starting with the experiment shown in Figure 1 and with 2–3 control CMGs performed between the different groups of CMGs to determine the recovery of the bladder reflex. In each group of CMGs, three CMGs were performed in sequence: (1) during left PNS, (2) during right PNS, and (3) during bilateral PNS. To determine the interaction between left and right PNS, different combinations of PNS intensities (1T or 2T) were tested in the 4 groups of CMGs: group 1 – left 1T and right 1T (Figure 1, *N* = 10 cats); group 2 – left 1T and right 2T (Figure 2, *N* = 8 cats); group 3 – left 2T and right 1T (Figure 3, *N* = 6 cats); group 4 – left 2T and right 2T (Figure 4, *N* = 8 cats). A 5-min rest period was inserted between the repeated CMGs to allow the bladder to recover from contractions.

### Data Analysis

The control bladder capacity for each animal was determined by averaging the 2–3 CMGs prior to PNS in each test group. Then the bladder capacities measured before, during, and after PNS in each test group were normalized to the control capacity in order to minimize the large variations of the control capacities between individual animals. The data were averaged across animals under the same conditions. The results are presented as a mean ± standard deviation. Statistical significance (*p* < 0.05) was determined by repeated measures one-way ANOVA followed by Dunnett’s multiple comparison after successfully testing the data for normal distribution by KS normality test.

## Results

Pudendal nerve stimulation at 1T intensity did not significantly change the control bladder capacity (14.1 ± 4.3 mL, *N* = 10 cats) when it was applied either unilaterally or bilaterally (Figure 1). The control capacity was not changed after stopping PNS (Figure 1) and was maintained at the same level throughout the experiments. Please note that there was no carry-over effect of PNS observed in this study or previous studies (Larson et al., 2011; Kadow et al., 2016).

Unilateral PNS at 2T intensity significantly (*p* < 0.05) increased bladder capacity by 29.3 ± 25.5% (right PNS, Figure 2) or 28.8 ± 16.1% (left PNS, Figure 3). There was no difference between the inhibition induced by right and left PNS. This inhibitory effect was not changed by the ineffective contralateral PNS at 1T intensity (Figures 2, 3). However, bilateral PNS at 2T intensity significantly (*p* < 0.05) increased bladder capacity by 61.6 ± 37.8% indicating an additive inhibition induced by the effective left and right PNS (Figure 4). After stopping repeated PNS, control bladder capacity and the bladder contraction (amplitude and duration) were not significantly changed.

## Discussion

This study in anesthetized cats suggests that pudendal neuromodulation on the left or right side inhibits reflex bladder activity by independent mechanisms. An ineffective unilateral neuromodulation at 1T stimulus intensity which elicits an anal twitch response does not enhance the inhibitory effect of 2T contra-lateral neuromodulation (Figures 2, 3) or act synergistically with ineffective 1T contra-lateral neuromodulation to unmask inhibition (Figure 1). Furthermore, effective 2T unilateral neuromodulation only elicits an additive inhibitory effect to that induced by contra-lateral 2T neuromodulation (Figure 4). Stimulation on the left and right sides produced similar reponses. These results indicate that the suppression of reflex bladder activity by electrical stimulation of afferents in the pudendal nerve occurs primarily ipsilaterally and that a synergistic or supra-additive interaction of these independent inhibitory mechanisms induced by bilateral PNS would contribute relatively little to the efficacy of pudendal neuromodulation.

Pudendal nerve stimulation at 1T intensity did not significantly increase bladder capacity in this study, which could be due to the weak intensity (1T) that failed to activate the pudendal afferent fibers. However, this possibility seems unlikely because at 1T intensity the PNS induced observable anal twitching. It is known that anal and vaginal sphincter twitching can generate pudendal afferent firing in cats by motor-sensory coupling (Lagunes-Córdoba et al., 2010). Therefore, even if the 1T PNS only activated the motor fibers in the pudendal nerve the sensory fibers would also be activated by sphincter contractions. Therefore, it is reasonable to believe that the failure of 1T PNS to significantly increase bladder capacity in this study is due to a weak activation of pudendal afferents. However, bilateral pudendal afferent inputs induced by 1T PNS failed to produce a significant increase in bladder capacity (Figure 1), indicating that simultaneous stimulation of weak bilateral afferent inputs in an attempt to augment or potentiate the effect by convergent inputs may not be an effective strategy for enhancing pudendal neuromodulation. In addition, the pudendal afferent input induced by 1T PNS did not enhance the inhibitory effect induced by 2T PNS (Figures 2,3). If these results are translatable to clinical situations where the unilateral pudendal neuromodulation fails, it is likely that bilateral stimulation with a failed stimulation on one side will not produce any greater effect than the effective contralateral pudendal neuromodulation alone. Consistent with this conclusion, a previous clinical study (Marcelissen et al., 2011) showed that in patients where the unilateral sacral neuromodulation therapy failed, bilateral sacral neuromodulation produced significant improvement compared to the baseline only when the contra-lateral sacral neuromodulation alone produced significant improvement over the baseline.

Furthermore, clinical studies (Scheepens et al., 2002; van Kerrebroeck et al., 2005) did not find a significant difference between unilateral and bilateral sacral neuromodulation, although bilateral sacral neuromodulation was proposed previously for OAB treatment (Hohenfellner et al., 1998). The only beneficial effect of bilateral sacral neuromodulation versus unilateral sacral neuromodulation was a higher success rate during the stage I trial period (Pham et al., 2008). This increased success rate could be due to the additive effect from the second electrode rather than a synergistic interaction between the bilateral neuromodulation, because it is known clinically that when unilateral sacral neuromodulation fails, addition of second stimulating electrode on the contra-lateral side can be effective in some patients (Marcelissen et al., 2011). However, our current study in cats shows that bilateral pudendal neuromodulation can produce additive inhibition on reflex bladder activity (Figure 4). Whether bilateral pudendal neuromodulation is different from bilateral sacral neuromodulation still needs to be tested in a clinical trial to determine if an additive effect can be achieved.

The additive inhibitory effect induced by bilateral pudendal neuromodulation is probably occurring in the central nervous system instead of in the bladder muscle. This speculation is supported by recent studies in cats showing: (1) that under normal conditions during saline distention of the bladder sympathetic afferent activity in the hypogastric nerve induced by PNS does not produce sufficient activation of β-adrenergic receptors in the bladder muscle to inhibit reflex bladder activity (Kadow et al., 2016); and (2) that single neuronal activity on the right side of the sacral spinal cord induced by saline distention of the bladder can be significantly inhibited by PNS on the right side (Yecies et al., 2018). In future experiments it will be important to determine if this inhibition also occurs during contralateral PNS and if bilateral PNS elicits inhibition of greater magnitude. These electrophysiological experiments would directly evaluate our speculation regarding the mechanism underlying the additive effects of PNS. Whether the additive pudendal inhibition occurs in the brain including the pontine micturition center is also an interesting question to be explored in the future.

It is worth noting that the PNS intensity effective in inhibiting bladder activity is different for humans and cats. In human PNS intensity slightly below motor threshold is effective, but in cats the intensity is higher than motor threshold. This difference could be due to the fact that neuromodulation is applied in human under awake conditions, but it is applied in animal studies under anesthetized conditions. However, this difference does not invalidate the results of animal studies as long as PNS activates the central inhibitory mechanisms. In anesthetized animals the large amplitude bladder contractions are mediated by reflex mechanisms and OAB symptoms in humans are mediated in part by involuntary reflex mechanisms. Thus, pudendal neuromodulation may act on the same central targets in both cases. In addition, saline was used in this study to distend the bladder, which activates reflex bladder activity mediated by non-nociceptive pelvic A-δ afferents. The interaction between bilateral pudendal neuromodulation could be further investigated using acetic acid to irritate the bladder, activate nociceptive pelvic C-fiber afferents, and induce bladder overactivity that may come closer to approximating the human OAB condition. However, the pathological causes of human OAB are currently unknown. Human OAB could be caused by neuronal overactivity in the micturition reflex pathways mediated by non-nociceptive pelvic A-δ afferents, nociceptive pelvic C-fiber afferents, or both. Our current study focused on the effects of pudendal neuromodulation on micturition reflex pathway mediated by non-nociceptive pelvic A-δ afferents.

This study in anesthetized cats reveals that an additive inhibition of reflex bladder activity can be induced by bilateral pudendal neuromodulation. This result indicates that bilateral pudendal neuromodulation might achieve better therapeutic efficacy in treating OAB than unilateral pudendal neuromodulation, thus providing basic science evidence to support clinical efforts aimed at improving pudendal neuromodulation therapy for OAB patients.

## Data Availability Statement

The raw data supporting the conclusions of this article will be made available by the authors, without undue reservation, to any qualified researcher.

## Ethics Statement

The experimental protocol and animal use in this study were reviewed and approved by the Animal Care and Use Committee at the University of Pittsburgh.

## Author Contributions

All authors listed have made a substantial, direct and intellectual contribution to the work, and approved it for publication.

## Conflict of Interest

The authors declare that the research was conducted in the absence of any commercial or financial relationships that could be construed as a potential conflict of interest.
